# Supplementary material for: Neoteric Algorithm Using Cell Population Data (VCS Parameters) as a Rapid Screening Tool for Haematological Disorders
Source: Diagnostics (Basel). 2021 Sep 9;11(9):1652. doi: 10.3390/diagnostics11091652 (PMC8469496; doi:10.3390/diagnostics11091652)
Supplement: Supplementary file 1 [file diagnostics-11-01652-s001.zip › Supplementary S1 Description of CPD Parameters.pdf]

Parameter	Mean Ne	SD Ne	Mean Ly	SD Ly	Mean Mo	SD Mo	Mean Eo	SD Eo
**Volume**	@MN-V-NE	@SD-V-NE	@MN-V-LY	@SD-V-LY	@MN-V-MO	@SD-V-MO	@MN-V-EO	@SD-V-EO
**Conductivity**	@MN-C-NE	@SD-C-NE	@MN-C-LY	@SD-C-LY	@MN-C-MO	@SD-C-MO	@MN-C-EO	@SD-C-EO
**Upper Median Angle Light Scatter (UMALS) 20º-42º**	@MN-UMALS-NE	@SD-UMALS-NE	@MN-UMALS-LY	@SD-UMALS-LY	@MN-UMALS-MO	@SD-UMALS-MO	@MN-UMALS-EO	@SD-UMALS-EO
**Lower Median Angle Light Scatter (LMALS) 10º-20º**	@MN-LMALS-NE	@SD-LMALS-NE	@MN-LMALS-LY	@SD-LMALS-LY	@MN-LMALS-MO	@SD-LMALS-MO	@MN-LMALS-EO	@SD-LMALS-EO
**Low Angle Light Scatter (LALS) 5.1º**	@MN-LALS-NE	@SD-LALS-NE	MN-LALS-LY	@SD-LALS-LY	@MN-LALS-MO	@SD-LALS-MO	@MN-LALS-EO	@SD-LALS-EO
**Axial Light Loss (ALL) Aº**	MN-AL2-NE	@SD-AL2-NE	@MN-AL2-LY	@SD-AL2-LY	@MN-AL2-MO	@SD-AL2-MO	@MN-AL2-EO	@SD-AL2-EO
**MALS, (UMALS+LMALS)**	@MN-MALS-NE	@SD-MALS-NE	@MN-MALS-LY	@SD-MALS-LY	@MN-MALS-MO	@SD-MALS-MO	@MN-MALS-EO	@SD-MALS-EO

Footnote:

Abbreviations:

MN= Mean, SD= Standard Deviation, volume (V), conductivity (C), Axial Light Loss (ALL) Aº, Low Angle Light Scatter (LALS) 5.1º, Lower Median Angle Light Scatter (LMALS) 10º-20º, Upper Median Angle Light Scatter (UMALS) 20º-42º, MALS, (UMALS+LMALS), NE (neutrophil), LY (lymphocyte), MO (monocyte), EO (eosinophil).

Interpretation of the table:

MN-V-LY = Mean volume of lymphocyte

SD-V- LY = Standard deviation volume of lymphocyte

MN-MALS- LY = Mean of scatter angle MALS for lymphocyte

SD-MALS- LY= Standard deviation of Mean of scatter angle MALS for lymphocyte
